# A Glass of Brandy

**Published:** 1868-12

**Authors:** 


### "A GLASS OF BRANDY

Can't hurt anybody! Why I know a person, yonder he is now, on high change, a specimen of manly beauty, a portly six-footer. He has the bearing of a prince, for he is one of our merchant princes. His face wears the hue of health, and now, at the age of fifty odd, he has the quick elastic step of our young men of twenty-five, and none more full of mirth and wit than he, and I know he never dines without brandy and water, and never goes to bed without a terrapin or oyster supper, with plenty of champagne, and more than that, he was never known to be drunk. So here is a living exemplar and disproof of the temperance twaddle about the dangerous nature of an occasional glass, and the destructive effects of a temperate use of good liquors."

Now it so happened that this specimen of safe brandy drinking was a relation of ours. He died in a year or two after that of Chronic Diarrhœa, a common end of those who are never drunk, nor ever out of liquor. He left his widow a splendid mansion up town, and a clear five thousand a year, besides a large fortune to each of his six children; for he had ships on every sea and credit at every counter, but which he never had occasion to use. For months before he died—he was a year in dying—he could eat or drink nothing without distress, and at death, the whole alimentary canal was a mass of disease; in the midst of his millions, he died of inanition. That is not the half, reader. He had been a steady drinker, a daily drinker, for twenty-eight years. He left a legacy to his children, which we did not mention. Scrofula has been eating up one daughter for fifteen years; another is in the mad-house; the third and fourth of unearthly beauty, there was a kind of grandeur in that beauty, but they blighted, and paled and faded, into heaven we trust, in their sweetest teens; another is tottering on the verge of the grave, and only one is left with all the senses, and each of them is weak as water. Why, we came from the dissecting-room and made a note of it, it was so horrible——

A gentleman of thirty-five was sitting on a chair, with no specially critical symptom present, still he was known to be a "Dissipated young man" as the saying goes. He rose, ran fifty feet, fell down and died. The doctors see a beauty in death, the chance of cutting up a fellow and looking about for sights. The whole covering of the brain was thickened, its cavities were filled with a fluid which did not belong to them, enough to kill half a dozen men with apoplexy; a great portion of one lung was in a state of gangrene, and nearly all the other was hardened and useless; blood and yellow matter plastered the inner covering of the lungs, while angry red patches of destructive inflammation were scattered along the whole alimentary canal. Why, there was enough of death in that one man's body to have killed forty men. The doctor who talks about guzzling liquor every day, being "healthy," is a perfect disgrace to the medical name, and ought to be turned out to break rock for the turnpike for the term of his natural life at a shilling a day, and find himself.

